# Lead Compounds in the Context of Extracellular Vesicle Research

**DOI:** 10.3390/pharmaceutics12080716

**Published:** 2020-07-30

**Authors:** Thao T.D. Tran, Phuong H.L. Tran

**Affiliations:** 1 Institute of Research and Development, Duy Tan University, Danang 550000, Vietnam; trantdinhthao@duytan.edu.vn; 2 The Faculty of Pharmacy, Duy Tan University, Danang 550000, Vietnam; 3 Deakin University, School of Medicine, IMPACT, Institute for innovation in Physical and Mental health and Clinical Translation, Geelong, Australia

**Keywords:** small extracellular vesicles, exosomes, lead compounds, biogenesis, drug delivery systems

## Abstract

Studies of small extracellular vesicles (sEVs), known as exosomes, have been flourishing in the last decade with several achievements, from advancing biochemical knowledge to use in biomedical applications. Physiological changes of sEVs due to the variety of cargos they carry undoubtedly leave an impression that affects the understanding of the mechanism underlying disease and the development of sEV-based shuttles used for treatments and non-invasive diagnostic tools. Indeed, the remarkable properties of sEVs are based on their nature, which helps shield them from recognition by the immune system, protects their payload from biochemical degradation, and contributes to their ability to translocate and convey information between cells and their inherent ability to target disease sites such as tumors that is valid for sEVs derived from cancer cells. However, their transport, biogenesis, and secretion mechanisms are still not thoroughly clear, and many ongoing investigations seek to determine how these processes occur. On the other hand, lead compounds have been playing critical roles in the drug discovery process and have been recently employed in studies of the biogenesis and secretion of sEVs as external agents, affecting sEV release and serving as drug payloads in sEV drug delivery systems. This article gives readers an overview of the roles of lead compounds in these two research areas of sEVs, the rising star in studies of nanoscale medicine.

## 1. Introduction

Among all processes of drug discovery, the most important goal is the identification of the best lead compounds that are subsequently advanced to the development stage. A “lead compound”’ is defined as a chemical entity that has newly discovered promising therapeutic activity and into which further chemical modifications can be introduced to develop a new compound with optimized beneficial effects and minimized side effects [1]. Lead compounds have been used to develop new antimalarial [2,3], anti-inflammatory [4,5], anticancer [6,7,8], anti-platelet [9,10,11], and anticoagulant [12,13] agents. To transform these compounds for use as drug candidates in clinical trials, a number of requirements, such as stability, production with cGMP (current good manufacturing processes), identification of metabolic pathways and potential drug–drug interactions, toxicology assessments, and clinical development plans, must be met to establish a model pathway of drug development [14].

Recently, in the fruitful research areas involving exosomes, lead compounds have been exploited in studies of exosome biogenesis and secretion. Lead compounds have been identified from libraries of chemical compounds and by screening technologies [15]. High-throughput screening (HTS) has been established as an essential tool for mapping and identifying potential lead compounds [16]. In this method, robots, detection platforms, and software are utilized, and since 2008, HTS has enabled researchers to analyze 100,000 compounds per day [16]. Alternatively, the simultaneous exposure of billions of compounds to targets in DNA-encoded libraries (DELs), which are tightly linked to unique DNA barcodes to enable their identification, enables preferred efficiency levels and costs to be applied to classical HTS [17]. With regard to exosome studies, in the latest update from Théry’s group, the exosome was defined as a subtype of extracellular vesicle (EV) (50–150 nm) that is released from a cell upon the fusion of an intermediate endocytic compartment, the multivesicular body, with the plasma membrane; in contrast, the microvesicle is formed and released by budding from the plasma membrane of a cell, and microvesicles have an extensive size range (100–1000 nm in diameter) [18]. In sizes that range from 50 nm to larger than 1000 nm, apoptotic bodies constitute a type of EV. Small EVs (sEVs), which are a highly enriched fraction of exosomes [18], is a more appropriate term than exosomes; therefore, we use the term sEVs instead of exosomes in this review.

Given that exosome research is important for elucidating the mechanisms of intercellular communication, specifically targeted delivery and diagnosis, studies of exosome biogenesis and secretion, such as their pathways and the effects of agents on these processes, have been important foci. sEV markers play critical roles in studies of exosome biogenesis, normal physiology, disease pathogenesis, including applications in which they serve as pathophysiological sEV biomarkers for diseases such as cancer. The most common marker proteins in sEVs are surface tetraspanins (CD63, CD81, and CD9). In addition, exosomal proteins have been found as potential diagnostic markers in various tumors, namely a few typical ones: CEA (colorectal cancer) [19], Her2 (breast cancer) [20], Glypican-1 (breast/ pancreatic/prostate cancer) [21], PSA (prostate cancer) [22]. Furthermore, sEVs have increasingly become promising drug delivery vehicles because of their unique advantages, such as stability, long circulation system half-life, no inherent toxicity, and high human compatibility [23,24]. In addition, the sEV structure comprises an aqueous core and a lipid bilayer membrane [21], facilitating both hydrophilic and lipophilic drug loading into sEVs. Thus, sEVs are considered ideal delivery systems for biomedical applications. A variety of methods have been used to package cargo into sEVs, including simple incubation of cargo(s) mixed with sEVs [23], sonication for a few cycles with controlled on and off pulse times [24], transfection using cationic lipids [25], and electroporation by applying an electric field to a mixture of sEVs and cargo(s) [26].

In this review, we discuss the roles of lead compounds in EV research, particularly their impact on sEV biogenesis and capacity to be loaded into sEVs. External agents in the production process of sEVs are among the critical factors that can affect the release of sEVs. In addition, taking advantage of the topography of sEVs, smart state-of-the-art technology designs are expected to enable the loading of a variety of compounds that have different levels of water solubility. These two research orientations in EV studies are important for developing effective sEVs that can deliver drugs to desired sites as the next generation of nanosized drug delivery systems for use in treatments.

## 2. The Contributions of Lead Compounds to sEV Biogenesis

**Figure 1:**
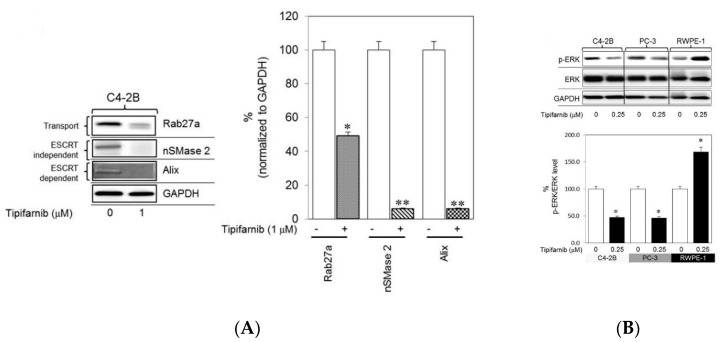
Investigation of the ability of tipifarnib to inhibit exosome biogenesis and secretion. Through the results of significant decreased levels of protein bands, immunoblot analysis shows (**A**) tipifarnib (1 μM) inhibited the protein concentrations of Alix, nSMase2, and Rab27a in C4-2B cells and (**B**) inhibition of the activation of pERK in C4-2B and PC-3 cells, but not in the normal prostate epithelial cell line RWPE-1. * *p*  <  0.05, ** *p*  <  0.01 compared to the controls. Figure was adapted from Datta et al. [47] with permission under the license http://creativecommons.org/licenses/by/4.0/.

Details of sEV biogenesis have been reported in many studies and reviews [25,26,27,28,29,30,31,32,33,34,35,36]. In brief, sEVs are produced when the cell membrane folds inwards to generate an early endosome. Inside the cell, a multivesicular body (MVB) is subsequently formed when the invaginated membrane also inwardly buds, giving rise to intraluminal vesicles (ILVs). This formation is the so-called late endosome. When the MVB is fused with the cell membrane, the vesicles which are released into the extracellular space are called exosomes (referred to as sEVs in this review). The formation of ILVs, and the processes of MVB formation, vesicle budding, and protein cargo sorting, requires the endosomal sorting complex required for transport (ESCRT) function [25,26,27,32]. Different ESCRT components (ESCRT-0, -I, -II, and -III) and ESCRT-associated proteins, such as Hrs, STAM1, TSG101, VPS4, ALIX, CHMP4, have been identified in sEVs secreted from cells of various lineages, supporting evidence that the ESCRT pathway is involved in exosomal biogenesis [32]. In addition, the biogenesis of sEVs can proceed in ESCRT-independent pathways, such as through ceramide-dependent mechanisms [37,38], oligomerization of oligomers [39,40], and tetraspanin-enriched microdomains [41,42]. Although the pathways have been commonly classified into ESCRT-dependent or ESCRT-independent types, they might be synergistically responsible for the mechanism of exosomal biogenesis. For controlling the secretion of sEVs, factors such as cell type, and cellular homeostasis are critical and should be considered [43]. Moreover, the number of sEVs released from cells and contents that the sEVs carry are influenced by culture conditions, external reagents, or any changes of environmental conditions. For instance, a reagent which affects levels of intracellular calcium may induce the release of sEVs in a concentration-dependent manner [44]. In contrast, a neutral sphingomyelinase 2 inhibitor may block the release of sEVs [45,46]. Lead compounds, thus, have been studied to investigate its capability of stimulating sEVs release [47]. Other factors which have been reported to have a modulation in sEVs release are heparanase [48], irradiation [49], oxidative stress [50,51]. These factors not only can alter the levels of sEVs but also can affect the expression of biomarkers of sEVs, which have been identified as promising diagnostic tools for several diseases [52,53,54]. Recently, specific markers of sEVs have been investigated for their effectiveness in screening potential lead compounds that impact sEV biogenesis, release, and/or uptake. These compounds can be identified through a quantitative high-throughput screen (qHTS) assay. Different from the traditional HTS, which only tests one concentration of a compound that may give false positive or negative results, the qHTS can generate over 1000 profiles of concentration-response to screen compounds that may have adverse effects with precise, refractory manners to a variety of sample preparations [55,56]. qHTS via curve fitting and cheminformatics software provides data such as half maximal effective concentration (EC50), maximal response, Hill coefficient (nH), enabling the assessment of nascent structure activity relationships (SAR) [56]. Thus, the qHTS assays are important to identify the active compounds’ potencies for downstream applications with reliable biological activities. For instance, by using this technique, lead compounds that are able to restrain the release of cancer derived sEVs would be sorted out, making a valuable contribution to studies of the disease progress and cancer therapies [47]. In addition, the activity of lead compounds in modulating the secretion of sEVs can be evaluated using immunoblot analysis to record the expression of proteins involved in the ESCRT-dependent and ESCRT-independent pathways such as Alix, nSMase2, tetraspanins. To evaluate the secretion of these sEVs, particle size analyses are also conducted to demonstrate the information of diameter, distribution, and concentration of the released sEVs. More details of the released sEVs with respect to their purity, specificity, and the proteins inside and on the surface can be explored as well, by using flow cytometry. Datta et al. screened lead compounds that regulate the biogenesis and release of sEVs from CD63-GFP-expressing metastatic castration-resistant C4-2B prostate cancer (PCa) cells [47]. They found that tipifarnib, neticonazole, climbazole, ketoconazole, and triadimenol were potent inhibitors and that sitafloxacin, forskolin, SB218795, fenoterol, nitrefazole, and pentetrazol were activators of biogenesis and/or secretion of PCa cell sEVs. By suppressing Alix (an ESCRT-dependent protein), nSMase2 (an ESCRT-independent protein), and Rab27a (involved in protein transport) using ketoconazole or tipifarnib, multiple pathways through which biogenesis and secretion of sEVs were commensurately suppressed. For instance, tipifarnib, with the capability of inhibiting cell growth and inducing apoptosis, was determined to have definitive inhibitory effects, even at small concentrations (in the nanomolar range) [47]. This compound inhibits the biogenesis and secretion of sEVs via both ESCRT-dependent and ESCRT-independent pathways. The expression of Alix, nSMase2, and Rab27a was significantly decreased in C4-2B cells treated with tipifarnib (Figure 1A). In addition, based on previous reports indicating that a farnesyl transferase inhibitor similar to tipifarnib decreased Ras activation and ERK phosphorylation [57,58,59], tipifarnib was hypothesized to disrupt Ras/Raf/ERK signaling pathways, a supposition supported by the significant decrease in pERK levels in both C4-2B and PC-3 (human prostate cancer epithelial metastatic cell line) cells treated with tipifarnib (Figure 1B).

The literature describes a mechanism by which lead compounds, such as azole groups, may inhibit the release of sEVs by reducing cholesterol synthesis because of their pharmacological action on the cross-inhibition of mammalian CYP51 [60]. In addition, another mechanism would be the regulation of Phase-II aldehyde dehydrogenase (ALDH) enzymes as ALDH is a Phase-II drug-metabolizing cytosolic enzyme [47]. These azoles have been suggested to be anticancer agent candidates worthy of further investigation [47]. Indeed, Gu et al. explored the effect of neticonazole, an sEV secretion inhibitor, as a therapeutic agent in suppressing intestinal dysbacteriosis (IDB, disruption of gut microbiome), which induces tumorigenesis and leads to colorectal cancer (CRC) [61]. They studied whether the production of sEVs was affected by IDB using mice bearing CRC xenograft tumors from the SW480 cell line. Blood from these mice was collected to investigate the secretion of sEVs in serum. Their study indicated that neticonazole inhibited the promotion of IDB in tumor growth and the production of sEVs and, interestingly, greatly enhanced the survival of tumor-bearing IDB mice, likely due to the capability of neticonazole to induce apoptosis of the CRC tumor cells. On the other hand, scientists have suggested using non-toxic agents to study their impacts on exosome production, specifically to ensure that less than 5% of the cultured cells die, thus minimizing the apoptotic potential of the EVs harvested in the supernatant of these cultures [62,63,64]. Researchers may employ a nonlethal compound that is identical to a lead compound; for instance, instead of using the lead compound 3-(5-methoxy-2-methyl-1H-indol-3-yl)-1-(4-pyridinyl)-2-propene-1-one (MOMIPP), MOPIPP (the methyl group on the 2-position of the indole ring is replaced by a propyl group) was used to explore the effects on exosome secretion [65]. This study demonstrated that glioblastoma cells treated with MOPIPP induced exosome biogenesis and/or secretion, as proven by the vacuolization promoted in the late endosomal compartments and the significant increase in the release of exosomal marker proteins (CD63 and Alix).

## 3. The Application of Lead Compounds as Drugs Loaded in sEVs

In the early stages of drug discovery, natural sources were valuable to produce lead compounds through a series of isolation and purification methods, HTS, biochemical and pharmacological tests, safety tests, and pharmacokinetic tests before the drugs were entered into clinical trials [66]. Compared to synthetic therapeutic agents, natural products exhibit efficient therapeutic activities with minimal side effects; engage in more interactions with proteins, enzymes, and other biological molecules and fewer with heavy metals; show greater molecular rigidity [66]. Many predominantly used compounds are semisynthetic drugs, a hybrid of natural and synthetic sources. Historically, morphine, isolated from the opium poppy (*Papaver somniferum*), was the first plant-derived drug [67], and aspirin was the first semisynthetic drug synthesized based on salicin, a natural compound isolated from *Salix alba* [68]. Subsequently, many new chemical identities were discovered and developed to establish the lead compounds that have been used to date. Table 1 summarizes lead compounds based on the history of their entirely/partially natural development; some compounds on this list are still among the most important drugs in the healthcare sector and research field.

Lead compounds during the drug discovery process have poor/extremely low water solubility [69,70,71,72]. In the early stage of the drug development process, pH adjustment and salt formation were the two most commonly used conventional approaches for formulating drugs with improved water-solubility [73,74,75,76]. In addition, amorphous dispersions of drugs and polymers obtained from spray-drying and hot-melt extrusion techniques and polymeric nanoparticles were also two major strategies to deliver these kinds of drugs [77,78,79]. In recent studies, lead compounds have been employed in a newly emerging delivery system using sEVs. Drugs are commonly loaded into sEVs by passive loading, incubation, or active loading methods based on sonication, extrusion, freeze-thaw cycles, electroporation, and incubation with membrane permeabilizers [80,81]. With these methods, drugs can be added in a pre-loading step by endogenous loading, in which the drugs are introduced in the sEV production stage, or they can be post-loaded with exogenous loading, in which the drugs are loaded after the isolation and purification steps of sEV production. Notably, sEVs have a shell composed of a lipid bilayer membrane and an aqueous core [82,83]. Thus, hydrophilic drugs are likely to localize to the aqueous core, whereas hydrophobic drugs tend to occupy the sEV membrane. Some factors that can prevent high levels of drug loading should be considered during the loading process. For instance, sEVs are already occupied by several proteins, DNAs, mRNAs, and miRNAs, which may leave little space in the small total available area of the sEV membrane for an external agent to embed [84]. The sEVs before or after drug loading are commonly collected by ultracentrifugation (UC), density gradient centrifugation, size exclusion chromatography (SEC), and ultrafiltration. The detailed description of each method, showing its advantages and disadvantages, can be found in many reviews and studies [85,86,87,88,89,90,91,92] and is not within the scope of this review. However, it should be noted that a combination of sEV isolation methods on occasions would be the ideal way to obtain an optimized high yield of purified functional sEVs [93]. In addition, the use of combined drug-loading methods would be useful to maximize the amount of drug loaded into sEVs [84]. An achievement in purified sEVs carrying a high drug loading content is expected in exosomal drug delivery systems, and particularly crucial in certain cases associated with specific diagnosis and therapies, for example, in a highly expected future clinical application of sEVs as effective delivery systems [94]. These sEVs are important to attain not only an optimum dose frequency but also improved therapeutic outcomes as a targeted drug delivery system [95]. Especially, these purified sEVs will be of great importance if sEVs are isolated from a patient for diagnosis and monitoring of the disease during the treatment. Moreover, assuming these sEVs will be transferred back to the same patient after subjected to some in vitro modifications including drug loading process and/or target engineering, these sEVs should be obtained as purified sEVs with high loading capacity for a precision at the targeted disease site to achieve the best effective treatment and avoid unwanted side effects.

Recently, with the development of the EV research field, one of the most promising applications for drug delivery involves natural lead compounds being loaded with cargos for use as drug delivery systems. To optimize the loading efficiency, researchers in the field have used sEVs collected at pre-isolation or post-isolation step to encapsulate external cargos. In the former approach, parental cells are treated with the loading cargo which is then released in sEVs and collected as the cargo-loaded sEVs in the isolation process of sEVs [96,97]. This approach is relatively simple and does not require further steps following common procedures of sEVs isolation. However, it has disadvantages such as low loading efficiency, possibilities of cytotoxicity of the loading cargo to cells [97]. Particularly, this method is useful and preferably selected to load oligonucleotides or proteins, though one relevant technical issue that should be considered is the possibility of protein degradation in parental cells. To overcome this issue, Batrakova et al. suggested using nanoparticles cross-linked with an excess of a non-biodegradable linker to protect catalase before loading into sEVs [96,98]. In addition, strategies that have been developed to modify sEVs after their isolation are also crucial for further applications. For instance, one of the common methods is electroporation, in which an electric field is applied to the suspension of sEVs and pre-selected cargos such as small molecules or oligo-nucleotides [99,100]. This intervention created pores in the lipid membrane of sEVs and hence, facilitating the loading of cargo into the sEVs. Another approach facilitating the membrane permeability of sEVs is using a detergent, e.g., saponin, as a chemical modification of sEVs to efficiently load external cargos [97,101]. To optimize the surface of sEVs and improve the capability of sEVs as an effective delivery of both hydrophobic and hydrophilic cargos, recently researchers have innovated a strategy to engineer sEVs by fusing the lipid bilayer membranes of sEVs with liposomes using the freeze-thaw method [102,103]. This attempt has been suggested to reduce immunogenicity as well as to increase colloidal stability and half-life of sEVs in blood. The post-isolation approach has been likely used more commonly to load drugs like lead compounds, which is applied in typical works presented below.

The incubation approach is the first and most common method of drug loading considered because of its simplicity and convenience. The incubation time varies for each compound. For instance, only 5 min of incubation at 22 °C is needed to complete the loading process of curcumin into sEVs [104,105], whereas at the same temperature, paclitaxel (PTX) requires 1 h to be efficiently loaded into sEVs [106]. In another study, PTX was incubated for the same period (1 h) but at 37 °C with shaking [107]. Using the same method, incubation, and compound, a large difference in loading efficiency was shown in the two studies, specifically, an average of 9.2% of the cargo was loaded in the former study [106] and 1.44% was loaded in the latter study [107]. In addition to different temperatures, the cell origins from which the sEVs were derived differed: LNCaP and PC-3 PCa cell lines and RAW 264.7 cells [106,107], respectively. Thus, parental cell lines and temperatures are factors that affect the loading efficiency of a compound into sEVs.

**Figure 2:**
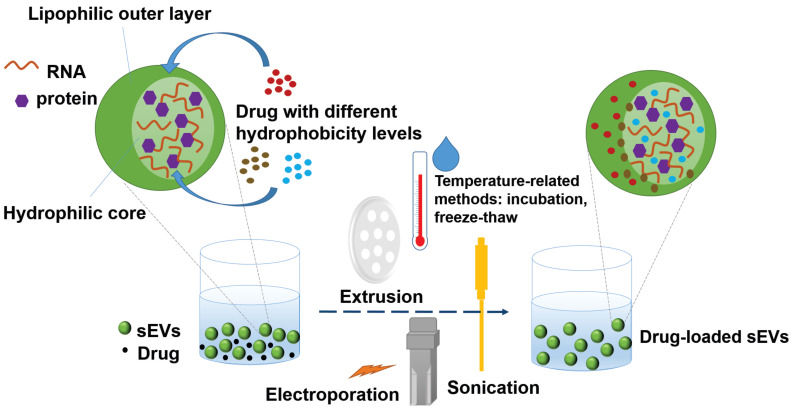
A typical loading process of lead compounds into small extracellular vesicles (sEVs) based on the hydrophobicity level of the drug and the loading method (active or passive).

Furthermore, as several previous reports indicated [97,108,109,110,111,112], different loading methods and different hydrophobicity levels of the compounds to be loaded play important roles in determining the loading efficiency of the compounds into sEVs. Figure 2 demonstrates the methods of loading drugs with different hydrophobicity levels into sEVs and their preferable localizations in sEVs based on their hydrophobic-hydrophilic properties. For example, in addition to the incubation method described above, PTX was also loaded into RAW 264.7 cell-secreted sEVs by the electroporation method and sonication method, and the loading efficiency was 5.3% and 28.29%, respectively [107]. Haney et al. loaded two different drugs, PTX and doxorubicin, into RAW 264.7 cell-derived sEVs [113]. As these two drugs have different hydrophobicity levels, the loading strategies were adjusted to achieve the best drug-loading efficiency. In the case of hydrophilic doxorubicin, different pH levels were reached during the incubation, with both sonication and incubation completed at room temperature. The pH was adjusted to decrease the charge of the molecule and increase the hydrophobicity of the drug. In contrast, the hydrophobic PTX was dissolved in ethanol and then evaporated to generate a thin film prior to incubation with the sEVs or directly added to the sEVs in a solution state during incubation [113]. PTX was prepared with the sonication approach as it had been with the incubation approach, except that the mixture was maintained on ice during sonication or was cooled only during the “off” cycles of sonication. The former procedure resulted in a lower drug-loading amount than the latter, suggesting that temperature is a critical factor affecting the drug-loading process. It was hypothesized that the sEV membranes are less rigid at room temperature than they are at a lower temperature, thus facilitating drug permeability into the sEVs [113]. Thus, the study suggested that the addition of the hydrophobic drug in its solution state to the sEV suspension be conducted at room temperature and that the sonication approach can achieve a higher drug-loading efficiency. On the other hand, another poorly water-soluble lead compound, triptolide, was dissolved in DMSO and loaded in SKOV3-derived sEVs by ultrasonication because it is mostly dissolved in this solvent [114]. Another strategy was proposed to enhance drug encapsulation efficiency of the sEVs by transforming drug hydrophobicity to amphiphilicity. In this way, the hydrophilicity-hydrophobicity of the drug drives the drug to both hydrophilic and hydrophobic sections of the sEVs. Aspirin had been formulated with poloxamer 407 and D-α-tocopherol polyethylene glycol 1000 succinate (TPGS) in a solid dispersion that was subsequently dispersed and accumulated in the sEVs to form a nano-amorphous matrix encapsulated sEVs through a combination of incubation, ultrasonication, and freeze-thaw methods [84]. These sEVs displayed enhanced cytotoxicity, efficient eradication of cancer stem cells, and promising targeted delivery in tumor models in vivo [115]. The membrane integrity of the sEVs frequently needs to be compromised during the process of active drug loading to ensure that the drugs can diffuse into the sEVs. This deformation process does not significantly affect the membrane-bound protein or the lipid content of the sEVs. Membrane integrity can be restored within an hour for sEVs that are incubated at 37 °C, probably owing to a number of consecutive processes including a reorganization of exosomal membranes, restoration of membrane microviscosity [107]. Other recently updated loading methods involve engineering sEVs with pH-sensitive reagents [116] and/or cationic lipids [117] or involve fusion with liposomes [103] or sEV-coated metal–organic framework nanoparticles [118]. The continuing development of methodological strategies for exogenous cargo loading will widen the application of therapeutic sEVs in diseases other than cancer.

## 4. Challenges in Lead Compound-sEVs Research

Prior to applying the lead compound-sEVs research into clinics, scientists in the field have encountered several challenges to ensure the quality and quantity of these sEVs is as high as possible. The production of sEVs from cell culture is apparently influenced by external factors such as environmental conditions or pharmacological treatments. Levels of this influence are extended to the number of sEVs collected from the cultured cells and cargoes of these sEVs. The sEVs secreted from body fluids such as blood, saliva, cerebrospinal fluid are not exceptions. Their concentrations and contents are varied by different diseased cells. For instance, the circulating sEVs are elevated in blood of cancer patients compared to those in healthy subjects, and the presence of tumor-specific proteins have also been found in these tumor-derived sEVs [20,119,120], and are likely modulated by the therapeutics and/ or by the variation of the cargo of sEVs which commonly occurs during progress of the disease [54]. Similar observations have been reported with sEVs collected from HIV patients who were under treatment of antiretroviral drugs, or from patients who suffered from acute injuries associated with lung, kidney, or myocardial [121,122]. Since these circulating sEVs play critical roles in predicting and monitoring responses to the treatment or disease progress, all of the external factors influencing the release of sEVs and their contents are extremely important and should be considered for investigation and evaluation to determine which is/or are the main factor(s) inducing the alterations. Lead compounds, thus, are one of the objects for the related studies and may or may not alter the production of sEVs. In other words, the role of lead compounds in sEV secretion may be varied in different cells. For instance, manumycin A can inhibit sEV biogenesis and secretion from castration-resistant prostate cancer (CRPC) C4-2B cells, but not normal RWPE-1 prostate cells [123]. In another context, contradictory results were reported between two research groups on the role of sulfisoxazole in inhibiting or not inhibiting the secretion of sEVs [124,125]. To this end, identification of lead compounds with optimal chemical and pharmacological properties is a critical challenge. Another issue after a successful identification of a new compound is that scientists need to select the most relevant in vitro assay to ensure an in vivo effective translation. In addition, despite the interest in using lead compounds to affect the production of sEVs, the possible cytotoxicity of such compounds is another concern as thorough understanding about their impacts on sEV biogenesis is lacking. A prerequisite for subsequent isolation and analysis of sEVs is that cell viability should be not less than 90%, to the presence of minimize apoptotic bodies in sEV collection [63]. The discovery of lead compounds which can either inhibit or stimulate sEV production without interfering the growth or viability of cells would be highly regarded.

Studies of sEVs as delivery vehicles of lead compounds have also been facing certain challenges in spite of their promising nanocarrier role. First, the cell origins should be in high attention to avoid any unwanted effects on responses to the drug delivery systems or unknown properties which may occur during the delivery. For instance, the possibility of oncogenic content of sEVs derived from cancer cells is one of the measures to consider clinical applications of these sEV types. Comprehensive characterizations of sEVs, thus, should be performed thoroughly prior to their application as therapeutic carriers. Optimized methods for isolation and purification of sEVs are also important to ensure that sEVs are the only objects to be collected for the targeting and delivery purposes of these nanovesicles. However, researchers in this field have been successful in obtaining the so-called “highly enriched” sEVs only. The presence of other types of vesicles in the EV family may result in misleading drug delivery applications or hinder efficiencies of the medication. In addition, the production of sEVs may be affected by manufacturing conditions such as scale-up performance, any changes of equipment or experimental conditions used for the production. For example, a selection of the isolation methodology (ultracentrifuge, ultrafiltration, size exclusion chromatography, precipitation, immune-affinity capture), or the use of ultracentrifuge equipment associated with the selection of rotor type (fixed angle or swinging bucket), centrifugal force, and ultracentrifugation time, have certain impacts on the total number and purity of harvested sEVs. Cvjetkovic et al. reported that excessive proteins were co-pelleted with sEVs beyond 4 h of ultracentrifugation; whereas, an insufficient recovery of sEVs was recorded with a 70 min-ultracentrifugation [126]. In addition, the selection of suitable type and number of culture flasks, and bioreactors (stirred-tank bioreactors employing microcarriers or perfusion-based production) should be carefully considered in large-scale production of sEVs [127]. All adjusted manufacturing conditions should not alter behaviors and properties of parental cells used for the sEV isolation. Cell culture conditions should be standardized for cell viability and tested for free mycoplasma contamination prior to the sEV isolation. This way ensures the sEVs are collected at high quality (purified sEVs), and the production is reproducible and maintains consistent properties of the extracted sEVs. Mycoplasma contaminants raise cautions in studies of sEVs on immunity since they were found to induce responses of B cells and not T cells, as well as induce splenocytes cytokine production [128,129]. Current technologies are limited to guarantee standardized and mass production of sEVs. Properties of sEVs can be varied and inconsistent even when they are isolated from the same parental cells. Thus, it is completely understandable that different properties and varied production of sEVs can be observed from different cells or different cell sources. Ultracentrifugation and sonication, two common techniques in isolation and drug loading of sEVs, are likely to create particle aggregation. Development of loading methods for encapsulating lead compounds into sEVs at high efficiency is also critical for effective treatments. Furthermore, to achieve the best therapeutic efficacy and avoid side effects, delivery of the lead compound-loaded sEVs should be accurate and efficient. Commonly, they are aimed to actively target cells (besides the naturally targeting behavior of sEVs) by an engineering of targeting ligands on the surface of sEVs, which requires highly sophisticated techniques to target specific cells [95,130,131,132]. For instance, to target the sigma receptor, which is overexpressed by lung cancer cells, Kim et al. engineered surface of PTX-loaded sEVs with aminoethylanisamide-polyethylene glycol (AA-PEG) vector moiety [95]. In another study, the surface-carboxyl superparamagnetic iron oxide nanoparticles were coated with A33 antibody to target A33-positive colon cancer cells and then, were bound to sEVs isolated from A33 positive LIM1215 cells loaded with doxorubicin [130]. Meanwhile, Wan et al. and Zou et al. decorated surfaces of sEVs with aptamers which were conjugated to a lipid-based linker to effectively tag the phospholipid bilayer of the sEVs based on a hydrophobic interaction [131,132]. More information on engineering targeting ligands on sEVs can be found in other reviews as the methodologies mentioned in these articles may be applied to lead compound-loaded sEVs [81,97,110,133]. The actively targeted sEV delivery systems, however, are still on their way to gain as many achievements as other research branches in the field [83]. Of note, while developing these techniques, how to produce the sEVs in a fast, convenient, and cost-effective manner is another challenge. New methods, hence, are in urgent demand to bring these promising delivery systems into clinical applications.

## 5. Conclusions

In the rapid expansion of sEV research around the world, sEV biogenesis and secretion are frequently the hot topics of study, as the production of sEVs provides valuable materials, often serving as the first resource needed to facilitate further downstream applications. Methodologies of sEV collection, isolation, and analysis undoubtedly contribute to the enrichment and reproducibility of sEVs. More importantly, as the ESCRT machinery mainly drives the process of sEV biogenesis, factors involved in cellular models and cellular homeostasis play important roles in sEV secretion. Specifically, to leverage the latter, a supply of external factors, such as irradiation or reagents (e.g., drugs), may cause cellular stress, which increases the secretion of sEVs. Lead compounds are emerging as new tools through which their effects on sEV biogenesis and secretion are studied. Although the employment of lead compounds in this research direction has just started, lead compounds show potential to shed light on the study of sEVs. A more detailed mechanism to explain why cells release more sEVs upon external stress has been under investigation. In addition, sEVs have been proven to potentially become the next generation of drug delivery systems for lead compounds. Although current studies are focused on anticancer compounds, strategies using the sEV-loading process show promise in terms of development and as prospects to be widely applied with different compounds in attempts to effectively target drug delivery systems in the research and development of new medications.

## Figures and Tables

**Table 1:** Important lead compounds originating naturally subjected to the drug development process and healthcare.

Lead Compounds	Natural Source	Pharmacological Activity
Artemisinin	*Artemisia annua*	Antimalarial
Aspirin	Semi-synthetic drug from salicin (*Salix alba*)	Analgesic, anti-inflammatory, (recently studied) anticancer
Berberine	*Berberis vulgaris L*.	Antidiabetic, anti-obesity, anticancer, anti-inflammatory
Caffeine	Coffee beans, tea leaves	Neonatal apnea, atopic dermatitis, treatment of Parkinson’s disease
Codeine	Semi-synthetic drug from morphine (*Papaver somniferum*)	Antitussive, analgesic
Colchicine	*Colchicum autumnale*	Anticancer, anti-inflammatory
Curcumin	*Curcuma longa L*.	Anticancer
Digoxin	*Digitalis lanata*	Cardiac glycoside
Doxorubicin	Semi-synthetic drug from cultures of *Streptomyces peucetius var. caesius* (soil fungus)	Anticancer
Morphine	*Papaver somniferum*	Pain relief, diarrhea
Nicotine	Tobacco	Anti-smoking
Noscapine	*Papaver somniferum*	Cough suppressant
Paclitaxel	*Taxus brevifolia*	Anticancer
Papaverine	*Papaver somniferum*	Vasodilator, gastrointestinal disorders
Penicillin	*Penicillium notatum* (fungus)	Antibacterial
Quinine	*Cinchona officinalis*	Antimalarial
Triptolide	*Tripterygium wilfordii Hook F*	Antitumor, anti-inflammatory, immunosuppressive
Vincristine and vinblastine	*Catharanthus roseus*	Anticancer
